# Production of a Health Awareness Video on Precautionary Measures Against Respiratory Infectious Diseases for Public Transportation Drivers: Protocol for a Scoping Review, a Delphi Study, and a Randomized Controlled Trial

**DOI:** 10.2196/89474

**Published:** 2026-06-19

**Authors:** Mahmoud Salam, Sleiman El Dhaybe, Leen Ghannam, Maya Serhan, Mira Hassan

**Affiliations:** 1 Alice Ramez Chagoury School of Nursing, Lebanese American University, Blat, Byblos, 10017, Lebanon, 961 70054397

**Keywords:** public transportation, respiratory infectious diseases, video, trial, Health Belief Model, cognitive dissonance

## Abstract

**Background:**

The public transportation system is a high-risk medium for respiratory infectious disease (RID) transmission. Health awareness videos are integral to disseminating knowledge, elevating health-risk perceptions, and enhancing attitudes toward precautionary measures against RID, as per the Health Belief Model and Cognitive Dissonance theory.

**Objective:**

This study aimed to produce and evaluate an evidence-based health awareness video on precautionary measures against RID among public transportation drivers (PTDs) in Lebanon.

**Methods:**

This is a multistage project executed over 2 fiscal years. In stage 1, a scoping review study identifies articles on RID among PTDs. Search strategy follows a combination of concepts (influenza, COVID-19, and public transportation) using PubMed, CINAHL Complete, and other databases up to September 2025. Studies that reported the characteristics of PTDs, their knowledge, perceptions, and compliance with precautionary measures against RIDs are reviewed. In stage 2, based on the scoping review, a script for a health-awareness video is drafted. Using a classic Delphi technique, the script is revised and approved by 8 experts in public health, infection control, psychology, and filmmaking, using the Patient Education Materials Assessment Tool for audiovisuals (PEMAT-AV), and DISCERN. PEMAT-AV (13-items) systematically evaluates the understandability and actionability of educational materials. DISCERN tool (16-items) evaluates the quality of consumer health information. In stage 3, a nonblinded, randomized, 2-arm controlled trial is conducted on 387 drivers from different regions. Using a simple randomization technique, the experimental group watches the new video. The control group watches a neutral video on traffic signs. To evaluate the effectiveness of the video, participants’ knowledge, risk perceptions, attitudes, and cognitive dissonance are measured using infection control guidelines, Health Belief Model tool, Vaccination Attitudes Examination scale, and dissonance thermometer. Using IBM SPSS, between-group analysis is performed with Student *t* test, ANOVA, and Pearson correlation, followed by forward-stepwise linear regression analyses (2-sided α=.025).

**Results:**

In stage 1, 20 relevant studies were identified. Studies from Lebanon described the sociodemographics, health- and work-related characteristics, and life challenges of the target population. Other studies reported influenza or COVID-19 vaccine hesitancy, low vaccine uptake, inadequate cleaning or disinfection of vehicles, and elevated risk perceptions among computers. The prevalence and impact of RIDs and determinants of precautionary measures against RIDs were identified. In stages 2 and 3, the video will be revised by experts (projected 5 months), produced (projected 6 months), and tested (projected 2 months). The study has not been funded yet.

**Conclusions:**

Findings from the scoping review study inspired the writing of the script (characters and dialogue), making it evidence-based and tailored toward the target viewers. A health awareness video on precautionary measures against RIDs contributes to a safer public transportation system and higher service usage. It aligns with the global mission to control the spread of RID and to lessen the disease burden.

## Introduction

### Background

Influenza and COVID-19 are highly contagious respiratory infectious diseases (RIDs) that rapidly spread in crowded enclosed settings, particularly during the cold months of the year [1]. The regional prevalence of RIDs in the Middle East ranges between 0.5% and 70%, contributing to notable morbidities and mortalities [2,3]. In Lebanon, a Middle Eastern country, laboratory-confirmed influenzas are linked to influenza types A (1631/2049, 76.9%) and B viruses (404/2049, 19.7%). COVID-19 surveillance system in Lebanon indicates that the overall cumulative mortality rate associated with COVID-19 is 119.6 per 100,000 individuals. The overall case fatality ratio is 1.3% [4]. The disease burden associated with influenza and COVID-19 in Lebanon is higher among older age groups, patients with obesity, and those with chronic medical conditions [5]. These figures necessitate raising public awareness on RID, especially among populations at high risk of contracting RIDs and those severely affected by its complications.

The public transportation system is known to be a high-risk media for RID transmission, especially during rush hours when public transportation drivers (PTDs) and users get in close contact with each other [6,7]. The risk of RID transmission surges with ridership and vehicle size [8]. RID transmission in public transportation systems is also a potential threat to the community at large. It severely affects children, older adults, chronic patients, pregnant women, and the immunocompromised. When PTDs get infected by RID, they become super spreaders [9]. Therefore, it is both an occupational and public health objective to promote safer public transportation systems [10].

A set of precautionary measures is recommended by the Centers for Disease Control and Prevention (CDC) to control the spread of RID, such as wearing facemasks, cough etiquette, hand hygiene, and social distancing [11-13]. Vaccination is highly recommended to lessen RID severity and shorten the duration of its infectivity [14]. Moreover, disinfecting high-touch surfaces inside public transport vehicles and at stations is also highly recommended to control RID transmission via surfaces [15]. All these measures disrupt the chain of infection transmission and promote a safer public transportation system [7,16-18].

### Benefits of Video-Based Health Education

Videos have been widely used in health awareness campaigns. Different formats include educational videos, patient testimonials or narrative videos, animation videos, short-form social media videos, live video streaming, virtual reality or immersive content, and video-based digital storytelling. Educational videos significantly improve health literacy and information recall compared with traditional text-based methods. Patient testimonials can improve the understanding of certain health conditions and provide emotional connection, yet they are relatively lengthier and limited by the considerable heterogeneity of the audience [19]. The effectiveness of video animations as information tools to deliver patient knowledge is inconclusive in the literature [20]. Live video streaming offers immediate real-time engagement with audiences, thus fostering stronger connections and trust, but it might not be convenient for PTDs whose job requires continuous or extended hours of driving [21]. Virtual reality with immersive content is commonly used as teaching or training methods, but it cannot be remotely implemented on broadly distributed populations like PTDs [22]. Digital storytelling can contribute to increased awareness, but concerns are centered on the accuracy and reliability of some of the information available online [23]. Therefore, short-form social media-based videos with educational content can be more suitable to PTDs.

Health awareness videos can enhance attitudes toward precautionary measures against RID and facilitate behavioral changes [24]. A systematic review of 28 studies showed that those videos are influential in promoting breast self-examination, prostate cancer screening, sunscreen adherence, self-care in patients with heart failure, HIV testing, treatment adherence, and others [25]. A study on COVID-19 health education videos circulated through WeChat (Tencent Holdings Limited), QQ (Tencent Inc), and other social media indicated that viewers are more likely to be compliant with precautions [26]. Health promotion videos can provide appealing, accurate, evidence-based, and comprehensible information that can resolve misconceptions or info-demics [27]. Videos can play an integral role in social learning and improve public health information [28].

### Previous Videos That Targeted PTDs

Videos on precautionary measures against RID that targeted PTDs on social media platforms were identified. Only 1 video from Lebanon was broadcast in April 2020 by a private taxi company during the COVID-19 pandemic as a marketing initiative [29]. Most videos were broadcast in western and regional countries. For instance, Dr Jack Caravanos from New York University posted 5 training videos on his YouTube (Google) channel in September 2021. His videos addressed taxi and rideshare drivers to inform them about COVID-19 and flu recommended safe work practices. They offered practical advice on facemask usage, vehicle sanitation, passenger seating protocols, and ventilation strategies [30]. Another video from the United Kingdom released in June 2020 demonstrated the installation of transparent protective screens in taxis and chauffeur vehicles to reduce COVID-19 transmission between drivers and users [31]. Featured on “صباح العربية” (Sabah-Alarabiya) in June 2020, a television media report on Egyptian taxi drivers portrayed the usage of various methods to protect both drivers and users from COVID-19 [32]. Furthermore, a video released by the Dubai Media Office in April 2020 showed Dubai taxi drivers implementing COVID-19 precautions, sanitizing their vehicles between trips, wearing masks, and installing protective partitions to reduce virus transmission [33].

### Characteristics of Health Awareness Videos

Public health officers interested in producing health awareness videos need to consider several criteria to ensure these videos have a meaningful impact. One study recommended first understanding the sociocultural features of the target audience and the context of the health problem being addressed, so that the video content is tailored and well-received by viewers [34]. This includes exploring the sociodemographics and health literacy of the target audience. Health awareness videos need to be relatively short and to the point, providing practical tips and clear recommendations on how to avoid health problems. They should project positive and motivational messages [35]. Videos can include images, animations, or real-life examples, with a clear and engaging narration, using the same spoken language and dialect of the target audience [36]. Health awareness videos should conclude with a take-home message to guide the target audience on the recommended actions. The source of the information needs to be added at the end of the video [37].

### Significance of the Study

PTDs and users might be unknowingly exposed to RID, so promoting their safety is a health priority [38]. If public transportation users feel safe during their ride, it contributes to higher service satisfaction and usage [39]. Using the public transportation systems reduces street congestion, controls pollution, saves money for users, and generates profits for stakeholders. Promoting a safe public transportation system reflects positively on public health. Promoting precautionary measures against RID aligns with the CDC mission to control the global spread of diseases and to lessen disease burden. Finally, health awareness videos are cost-effective, and they can reach a wider audience.

Many videos on social media platforms were broadcast 4-5 years ago during the COVID-19 pandemic, so in the absence of newly released videos, knowledge decay might occur [40]. Moreover, these videos neither showcased real-life scenarios featuring drivers and users nor did they present evidence-based information. The widespread accessibility to various social media platforms made the outreach of health awareness videos a rewarding cost-effective means of disseminating knowledge. Social media platforms are widely accessible in Lebanon [41,42]. In 2025, there were 5.34 million internet users in Lebanon (online penetration 91.6%). TikTok (ByteDance), YouTube, Facebook (Meta), and Instagram (Meta) social media platforms had 4.02, 3.19, 3.15, and 2.5 million users, respectively [43]. To the best of our knowledge, no recent videos were released lately in Lebanon despite the continuous spread of both influenza and COVID-19 in this country. Therefore, there is a need to deliver an updated, evidence-based health awareness video that captures the sociocultural perspectives of PTDs in Lebanon.

### Objectives

Guided by the Health Belief Model (HBM) and cognitive dissonance (CD) theory, the aim of this study is to produce and evaluate an evidence-based health awareness video on precautionary measures against RID among PTDs in Lebanon. HBM consists of 6 components, 2 of which are the perceived severity of and the perceived susceptibility to diseases [44-46]. CD is a psychological conflict that arises when a person holds inconsistent beliefs with a current behavior simultaneously [47]. Signs of CD include feeling uncomfortable, uneasy, bothered, disappointed, annoyed, disgusted, angry, ashamed, embarrassed, or guilty. It is hypothesized that when PTDs are exposed to a video on RID, they gain knowledge about it, experience an elevation in their health-risk perceptions, and demonstrate a positive attitude toward precautionary measures against RID. This video is likely to trigger a state of dissonance, but they will eventually rationalize with the importance of adhering to best practices and seek more information.

## Methods

### Overview

This multistage project is executed over 2 fiscal years. In stage 1, a scoping review study identifies articles on RID among PTDs. In stage 2, based on the findings of the review, a script for a health-awareness video is drafted. Using a classic Delphi technique, the script is revised and approved by 8 experts in public health, infection control, psychology, filmmaking, and public transportation. A video is then produced based on the approved script. In stage 3, a nonblinded, randomized, 2-arm controlled trial is conducted on 387 PTDs to evaluate the effectiveness of the video.

### Stage 1

A scoping review of literature guided by the PRISMA-ScR (Preferred Reporting Items for Systematic reviews and Meta-Analyses extension for Scoping Reviews) was conducted (Checklist 1). The aim of this review is to answer the following questions: (1) What are the sociodemographics, health-and work-related characteristics of PTDs in Lebanon? (2) What are their levels of knowledge and perceptions about RIDs? (3) What are the precautionary measures they follow against RIDs?

A library search strategy followed a combination of 4 broad concepts replicating the search strategy implemented in a scoping review study published in 2023 [48] (Table 1). Under each concept, specific medical subject headings were combined and searched in each database using common Boolean operators. The time frame for the published studies was up till September 30, 2025. The geographical space of the studies was limited to Lebanon, to ensure the script is socially and culturally relevant. Peer-reviewed articles indexed in PubMed, EMBASE, Scopus, CINAHL, Medline, and the Cochrane Library were screened. The full version of the articles published in subscription journals (restricted or paid access journals) was retrieved from the Joseph G Jabbra library at the Lebanese American University.

**Table 1.** Search terms.

Steps	Search terms
1	(influenzavirus AND a) OR (influenza AND a AND virus) OR (influenzavirus AND b) OR (influenza AND b AND virus) OR (influenzavirus AND c) OR (influenza AND human)
2	(coronaviridae OR (coronaviridae AND infections) OR coronavirus OR (coronavirus AND infections) OR betacoronavirus* OR betacoronavirus OR beta-coronavirus OR coronavirus* OR covid)
3	((sars AND virus*) OR (severe AND acute AND respiratory AND syndrome) OR sars OR sars-cov OR ((sars-associated OR sars-related) AND (cov OR coronavirus*)))
4	((middle AND east AND respiratory AND syndrome AND coronavirus) OR (middle AND east AND respiratory AND syndrome) OR (mers) OR (mers-cov) OR (mers-related) OR (mers-associated) OR (coronavirus) OR (cov))
5	((“2019” AND novel AND corona*) OR (“2019” AND new AND corona*) OR (“2019” AND cov) OR (“2019” AND ncov) OR (coronavirus AND disease) OR (coronavirus AND "2019”) OR (covid19) OR (covid-19) OR (novel AND corona*) OR (new AND corona*) OR sars2 OR sars-cov-2 OR (sars AND coronaviridae) OR (sars AND coronavirus) OR (sars AND coronavirus) OR ncov OR 2019-ncov)
6	1 OR 2 OR 3 OR 4 OR 5
7	((leban*) OR (liban*) OR (lubnan*) OR (lobnan*))
8	6 AND 7

### Stage 2

Using a classic Delphi technique, the script will be revised by a panel of experts in public health, infection control, psychology, filmmaking, and public transportation (drivers, users, and stakeholders). Delphi studies in social and health sciences are guided by the interdisciplinary standardized DELPHISTAR checklist [49] (Checklist 2). A purposive sampling method will be followed to identify these experts. The panel members will be reached out through their professional networks or based on their publication profiles, if they are experts in their field for at least 5 years. They will be invited by sending them a letter to their email addresses. They will be informed about the study objectives and the expected number of review rounds to obtain their approval and sign a confidentiality agreement form.

The expert in public health will ensure the script promotes good knowledge among viewers. The expert in infection control and prevention will advise on information regarding RID transmission, precautionary measures, risk factors, and vulnerable populations. The expert in psychology will emphasize the details in the script that would elevate health-risk perception, encourage positive attitudes, and influence behavioral changes. The expert in filmmaking will advise on the general plot, characters, duration, and the narrative text so that the health-awareness video leads to a meaningful impact among the target audience. A PTD will be involved in the review of the script to ensure the script mimics the real-life experience of drivers. A commuter will be invited to read the script and provide suggestions from an end user perspective of transportation services. A public transportation engineer can provide suggestions from a policymaker’s perspective. A public transportation company owner can provide suggestions from a business owner perspective.

Experts will objectively evaluate the script using the Patient Education Materials Assessment Tool – Audiovisual (PEMAT-AV) and DICERN tools. Additional subjective comments will be accommodated. Using the PEMAT-AV and DISCERN tools enhances the rigor of the script and establishes convergent validity. PEMAT-AV was developed by experts in health literacy, content creation, patient education, and communication [50]. PEMAT-AV systematically evaluates the understandability and actionability of educational materials. Understandability is the viewers’ ability to understand the video, regardless of their diverse backgrounds and varying levels of health literacy. Actionability is the viewers’ ability to identify what they can do based on the information presented in the video, regardless of their background and level of health literacy. PEMAT-AV produces separate numeric scores for understandability and actionability. PEMAT-AV consists of 13 items measuring understandability (3 statements do not apply to the script in this study, so they were removed) and 4 items measuring actionability (one statement does not apply to the script in this study, so it was removed). PEMAT-AV was designed to be completed by professionals, including health care providers and others [50]. A research team previously translated PEMAT-AV to Arabic language and performed cultural adaptation of this tool. The Arabic PEMAT-AV showed substantial reliability in the understandability domain (intraclass correlation coefficient=0.77, Cronbach α=0.87, but moderate reliability in the actionability domain (intraclass correlation coefficient=0.64, Cronbach α=0.78). Permission was obtained for the authors of the tool to use it in this study [51]. PEMAT-AV statements are rated either agree (score=1), disagree (score=0), or not applicable. The percentage mean score (PMS) will be calculated by summating the scores and dividing them by the total possible score. The statements of PEMAT-AV were customized by using terms relevant to the study objectives (PTDs, precautions against RID, and narrative script).

The DISCERN tool was developed by Charnock and her colleagues in 1999. DISCERN facilitates the production of new, high-quality, and evidence-based consumer health information. DISCERN became a reliable and valid instrument for judging the quality of written consumer health information. In a systematic review that measured the properties of online health information quality assessment tools, the DISCERN demonstrated Cronbach α coefficients above 0.7, indicating acceptable internal consistency, a high-weighted κ calculation, and a “very good” methodological quality rating [52]. It has been translated to numerous languages [53]. The DISCERN tool consists of 16 questions that are rated over a 5-point Likert scale. Out of 5 points, 1 means the content of the script did not address the precautionary measures against RID, 2-3 points mean the content partially addressed precautionary measures against RID, and 4-5 points mean the content adequately addressed precautionary measures against RID. DISCERN examines the ability of the script to clearly deliver the message on precautionary measures against RID to viewers. The total score ranges between 16 and 80, with intervals (low-quality: 16-32, moderate-quality: 33-64, high-quality scores: 65 and above) [54].

At least 3 rounds of review will be performed after circulating the script to all members of the panel. The first round will focus on exploring the characters, role plays, settings, and plot. Experts will evaluate the quality of health information integrated in the script. Suggested modifications will be carried out before the next round. If panel members have additional recommendations, they will be integrated in the script, and a third round of review will follow. In case one of the members did not respond at any round, the expert will be sent timely reminders. If nonresponse persists, the expert will be replaced by another expert with the same specialty. To encourage participation, the experts will be acknowledged in the future publication and video production. Nonresponses will be evaluated at each round to make sure the study objectives are still achievable [55]. Once consensus is reached, the video will be produced. Since some experts might prefer an Arabic language version of the DISCERN tool, it will be translated from English to Arabic language by 1 faculty and 1 registered nurse, then the Arabic version will be translated back by another 2 persons, both bilingual. This forward and back-translation will follow the World Health Organization–recommended process of instrument translation and adaptation [56] and revised for discrepancies. The final version of these tools will be revised for simplicity of its language structure and terms, comprehensible by a 12-year-old child.

Using IBM SPSS (version 30), the PEMAT-AV and DISCERN scores will be obtained from each member of the panel at every round of the review. For the PEMAT-AV scores, statistically significant changes in the understandability and actionability scores indicate that the quality of the script is enhanced with each round. The scores will be presented in means (SD). Paired *t* test (for 2 rounds of review) and repeated measures ANOVA (for 3 or more rounds of review if needed) will be performed if the conditions of normality are met. Otherwise, the Wilcoxon signed-rank test and the Friedman test will be used for the 2-round or 3-round review, respectively. For the DISCERN, scores will be converted to low, moderate, and high ability of the script to clearly deliver the message on precautionary measures against RID to viewers. DISCERN ordinal outcomes will be presented in frequencies and percentages for each round of review, and the Cochran Q test will be used to detect improvements in the clarity of the script across the review rounds. For all the tests, the level of significance is 0.025. While the change in scores indicates that the quality of the script is improving across the rounds of review, the decision to produce the video is based on the final approval of the panel members.

### Script of the Video

The script of the health-awareness video will include 5 main characters (a PTD, his wife, a second PTD, a commuter, and a commentator). The settings in the movie will include the PTD’s place of residency, street, transportation vehicle, and hospital. Characters in the script will be given locally recognized names. The sociodemographic of the main character, acting as a PTD, resembles the characteristics commonly reported in literature: middle age (48 y), male sex, work experience (11 y), married with children, striving to make a living, resides in a low socioeconomic area, smokes tobacco products, and did not receive influenza or COVID-19 vaccine. His young son is coughing, indicating the high chance of transmitting RIDs to his family members. The wife advises him on wearing a facemask while on duty, but he ignores its benefits and the seriousness of RIDs. He demonstrates fatalistic views when his friend (who is also a PTD) passed away due to RID. Throughout these scenes, a background commentator is emphasizing the following information:

- “PTDs are at high risk of contracting influenza and COVID-19” [7,16-18].
- “Every year, three to five million individuals worldwide get severely sick because of influenza. Between 250,000 and 500,000 individuals pass away” [2].
- “Influenza transmissibility in Lebanon is moderate, yet with a very high impact” [5].
- “A report from eight Lebanese hospitals showed that 30%‐40% of severe acute respiratory infections were due to influenza viruses. 20‐50 deaths per week due to influenza” [5].
- “More than one million confirmed cases of COVID-19 in Lebanon, among whom more than 10 thousand people died.” [4]
- “Work absenteeism (1.7%‐3.6%) among drivers due to influenza” [57].
- “Non-compliance rates with cleaning, disinfection, and deep cleaning (washing stations) in Lebanon is between 40.9% and 75.1%. Between 51.3% and 71.9% of drivers in Lebanon are not compliant with proper cleaning and disinfecting methods” [52].
- “62.1% of university students in Lebanon are concerned about the severity of RID, “50.6% feel susceptible to RIDs while using transport vehicles. 86.3% of students prefer if transport drivers clean/disinfect their vehicles” [51].
- “Commuters feel safer when drivers take precautions. A clean taxi isn’t just about COVID-19, it’s about customer satisfaction.” [58]
- “Taking precautions isn’t fear. It’s a responsibility”; “Study from Lebanon showed that drivers miscalculate the risk of RID. Some prefer natural immunity over vaccines. Vaccination is an essential primary healthcare objective with benefits at the individual, health care system, community levels.” “Vaccination allows for an expedited health recovery, fewer visits to clinics, less stress on healthcare systems, and lower medical bills” [59].
- “Vaccination shortens the duration of disease. A wide vaccine coverage promotes an efficient herd immunity, and subsequently less disease outbreaks” [60,61].
- “Risk of influenza transmission in passenger cars ranges between 59% and 99.9%, for a 90-minute trip” [8].
- “Crowdedness, unsanitary conditions, and close/prolonged interaction with passengers increase this risk” [6,7].

The script will end with a closing statement and the contact information of the Ministry of Public Health will show up for further assistance. The video will not contain any material that might offend, insult, degrade, or discriminate against study participants in any way possible. Medical jargon and complex terms will be avoided. The panel will ensure the narrative script is informative, simple, assertive, and clear.

### Stage 3

After the video is produced, a nonblinded, randomized, 2-arm controlled trial will be conducted on a group of PTDs, guided by SPIRIT (Standard Protocol Items: Recommendations for Interventional Trials) 2025 checklist [62] (Checklist 3). The experimental group will watch the newly produced health-awareness video once directly after giving consent, whereas the control group will watch a neutral video once about raising awareness about traffic signs in Lebanon [63]. The video assigned to the control group is short in duration (1:51 min), with soft background music, and displaying the universal traffic signs in Arabic language. The video was first released in 2020 aiming at raising awareness among motor vehicle drivers in Lebanon. After watching one of these videos in parallel, both groups will respond to a face-to-face questionnaire.

The Lebanese public transportation system consists of 33,500 exclusive or shared-ride taxis [64]. Public transportation vehicles are owned by 87,673 drivers who are registered in more than 20 different syndicates situated all over Lebanon (syndicates differ by the region, the type of service provided, and the category of vehicles) [65]. PTDs are eligible to enroll in this trial if they operate within the Lebanese public transportation system, regardless of their sex. Those to be excluded will be commercial drivers (trucks) or others who are not in daily contact with commuters. Drivers who are unwilling to enroll in the study or unable to comprehend the written or verbal Arabic language will be excluded. A proportionate sample of drivers will be invited to enroll in this study to obtain a representative sample of the whole target population, based on the distribution of licensed public transport vehicle owners across Lebanese regions (Table 2).

**Table 2.** Distribution and proportionate sampling of participants per governorates [65].

Governate	Sample (N=387), n (%)
Akkar Governorate	19 (5)
Baalbek-Hermel Governorate	19 (4.6)
Beirut Governorate	37 (9.6)
Beqaa Governorate	19 (4.4)
Keserwan-Jbeil Governorate	27 (7.1)
Mount Lebanon Governorate	189 (49)
Nabatieh Governorate	15 (3.9)
North Governorate	37 (9.5)
South Governorate	25 (6.9)

The trial study will be conducted in community settings where PTDs are naturally present (stations, parking lots, offices of syndicates, nearby businesses, academic, and tourist areas) situated in various Lebanese governorates (Beirut, Mount Lebanon, Jbeil-Kisserwan, North, Beqaa, and South). Sample size was calculated based on the influence of health-promotion videos on individuals’ attitudes and their effect sizes reported in the literature [47]. A systematic review of 54 studies from 18 different countries showed that health awareness videos exerted favorable influences on the individuals’ attitude (25/55, 45.45%), intention (29/55, 52.73%), and health-related behaviors, such as nutrition and vaccination (19/55, 34.55%). In total, 16 studies revealed a small to moderate overall effect size of g=0.33, *P*<.001, indicating a statistically significant effect of video-based health promotion on participants’ attitudes, while 15 studies produced a moderate overall effect size (g) of 0.41 (*P*=.01) [24]. Following Cohen guidelines, a conservative small to moderate effect size is reported in the literature (g=0.33). A significance level of 97.25% is considered to control Type I error (2-sided α=.025), and a power of 0.8 is considered to control type II error. The sample size is 352 that will be inflated by 10% (n=388) to compensate for any incomplete questionnaire or missing values [66]. Accordingly, the intervention and control groups will include 194 public transportation drivers each.

The principal investigator (M Salam) will train a group of senior nursing students on how to screen for PTDs, explain the study objectives, get informed consent, randomize the participants, invite them to watch the video, and then proceed with the questionnaire. Simple randomization technique will be performed by asking the participants to select a number from 1 to 10. Participants who select an even number will be assigned to the intervention group, while those who select an odd number will be assigned to the control group. Data collectors will be trained on responding to participants’ queries upon need. These nursing students have finished most of their academic courses and passed the Collaborative Institutional Training Initiative training.

After randomizing the study participants to intervention and control groups, study participants will be questioned about their sociodemographics (sex, age, place of residence, marital status, level of education, and financial status), their work-related variables (work duration in public transportation, type of transportation vehicle, and type of service provided), health-related variables (smoking, having a chronic medical disease, and vaccination). These variables are generally linked to health risk perceptions of respiratory pathogens, as per HBM [67-69].

Knowledge about the RID mode of transmission and prevention will be evaluated [67-69]. The PMS of knowledge scores (mean, SD) will be presented. Higher scores indicate better knowledge. This variable measures the effectiveness of the health awareness video. Participants’ health-risk perceptions will be evaluated following two dimensions of the HBM that are the perceived susceptibility (2 items) and the perceived severity of RID (3 items) [70]. The HBM tool was translated to Arabic language and adapted by a team of researchers from Qatar [71]. It will be slightly modified to meet the study context. Responses to risk perception will be rated over a 5-point Likert scale (1 strongly disagree to 5 strongly agree). The mean score of health-risk perceptions will be calculated. Higher scores indicate feeling more susceptible and fear of RID. This measures the effect of the health-awareness video on the participants’ feelings about being at risk.

Participants’ attitudes toward vaccination will be evaluated using 3 domains of the Vaccination Attitudes Examination (VAX) scale: unexplored side effects of the vaccines (5 statements), pharmaceutical companies’ profiteering from vaccines (3 statements), and preferred reliance on natural immunity (3 statements). VAX is a generic tool that identifies people with negative attitudes toward vaccines [72]. A group of Qatari researchers successfully adapted the VAX scale within the Arabic context (α=.78‐.93), and validation of the translated version was executed [71]. Each statement of the attitude dimensions will be rated over a five-point Likert scale that ranges from 1 (strongly disagree) to 5 (strongly agree). The scores of the statements will be summated and their composite scores will be presented (mean, SD).

CD is a self-reported measure of the emotional discomfort experienced due to a dissonance-triggering factor [73]. CD can be measured using the dissonance thermometer that consists of adjectives distributed across four indices (discomfort index, negative self-index, shame index, and positive index). All 4 subscales demonstrated acceptable levels of reliability, with the positive and negative affect scales exhibiting excellent internal consistency and test-retest reliability [74]. For this study, the discomfort index that consists of uncomfortable, uneasy, and bothered feelings will be used as adjective ratings (0 to 6). Higher scores indicate an increased level of discomfort due to the effect of the video content. More details about the scales and the schedule of assessments can be found in Table 3.

Data will be entered using an electronic survey through mobile devices. Data from anonymous questionnaires will be migrated from the electronic questionnaires into a Microsoft Excel sheet and then into SPSS. Responses will be coded and validated. Deidentified collected data will be stored within a secure server in an aggregate form. Each questionnaire will be coded anonymously by giving it an ID number. Participants’ data will remain anonymous during data entry or analyses. Errors in data will be controlled by setting limits for responses when building the electronic survey to avoid faulty entries. Nonresponse rate will be calculated by dividing the number of PTDs who refused participation by the total number of drivers who participated. Participants who fail to complete the survey (>30% missing responses) will be excluded and replaced with new participants within the assigned group (control or intervention) until the target sample size is met. The extent and pattern of missing data (random vs nonrandom missing values) will be evaluated and a decision will be made to impute for the missing values if <10% [75].

Data will be analyzed in the trial study by using SPSS (version 30). Sex, type of transportation vehicle, type of service provided, place of residence, marital status, level of education, and financial status will be described as categorical variables (n, %), whereas age and years in service will be described as continuous variables (mean, SD). Knowledge, perceptions, attitudes, and dissonance will be evaluated post intervention and compared with those obtained from the control group. Knowledge statements will be scored and converted to PMS. Risk perception, attitude statements, and dissonance will be scored, summated, and then converted to mean scores. These continuous variables will be tested for normality (transformed if skewed). The mean (SD) or median (IQR), if not normally distributed, will be used to describe continuous variables. Between-group analysis of mean differences will be tested using Student *t* test and 1-way ANOVA (for normally distributed data) and Kruskal-Wallis test with post hoc analysis (for skewed data). The relationship between continuous outcomes will be assessed using Pearson correlation analysis or Spearman ρ (for skewed data). Between-group analysis will indicate if the video being tested is effective in terms of raising awareness, elevating risk perceptions, enhancing attitudes, and triggering dissonance. Within-group analysis will identify subgroups or PTDs who are less responsive to the video content. Statistical significance will be at α<.025 (2-sided). After identifying the potential predictors of the study outcomes (bivariate analysis), forward stepwise linear regression analyses will be executed. This model identifies the percentage of variations in knowledge, risk perception, attitudes, and dissonance scores explained by each set of exposures. The increase in adjusted *R*^2^ will be compared in each step. Testing for any interaction or confounding effect of various exposures will be performed. Interim analysis will be performed by the principal investigator (M Salam) when the number of participants recruited is 40.

**Table 3.** Sequence of assessments.

Steps and variables	Measure
1	
Screen for eligibility criteria, explain study objectives, and obtain informed consent	—^a^
2	
Sociodemographics	—
Sex, place of residence, marital status, level of education, and financial status	n (%)
Age	mean (SD)
3	
Work-related variables	—
Type of transportation vehicle and type of service provided	n (%)
Work duration	mean (SD)
4	
Health-related variables	—
Smoking, having a chronic medical disease, and vaccination	n (%)
5	
Exposure to video (few minutes)	—
6	
Knowledge (dichotomous scale: 9 statements)	—
Knowledge about RID^b^ transmission or prevention	PMS^c^, mean (SD)
7	
Health-risk perceptions (5-point Likert scale: 5 statements)	—
Perceived susceptibility or severity to RID	MS^d^, mean (SD)
8	
Attitude toward vaccination (5-point Likert scale: 11 statements)	—
Unexplored side effects of the vaccines, profiteering from vaccines, and preferred reliance on natural immunity	MS, mean (SD)
9	
Cognitive dissonance (6-point Likert scale: 4 statements)	—
Discomfort index, negative self-index, shame index, and positive index	MS, mean (SD)

a Not applicable.

b RID: respiratory infectious disease.

c PMS: percentage mean score.

d MS: mean score.

### Ethical Considerations

The study was approved by the Institutional Review Board (IRB) at the Lebanese American University (LAU.SON.MS3.28/Apr/2026) on April 28, 2026. Study investigators will follow the recommendations of the International Conference on Harmonization for Good Clinical Practice, and the study will be in compliance with the Declaration of Helsinki. Changes in the study protocol will be reported to the IRB and updated on ClinicalTrials.gov. A self-explanatory letter of invitation will be provided by data collectors to eligible participants in the Arabic language. In case the content of the letter was not clear, data collectors will provide further explanation. Participants who agree to enroll will sign a written informed consent after acknowledging all the conditions pertinent to their participation. They will be informed that their participation may or may not benefit them directly, but it will provide them with new knowledge about the risks of RID at the individual and community levels. Participants will be informed that their contribution in this study will benefit public health officers. In case the driver decided not to participate or was not eligible, no further information will be collected. This study will not pose any risk or harm to study participants. Some drivers might feel uncomfortable after watching the video, so data collectors will be trained on how to remedy their concerns by raising awareness about precautionary measures, guiding them on sources of knowledge and places to obtain facemasks, hand sanitizers, and vaccines, in addition to relaxation techniques (deep breathing and distraction). These techniques can help individuals resolve emotional distress and set action plans to safeguard them against RIDs. The control group will be watching an educational video on traffic signs, thus not depriving them of any benefit. The decision to participate or not will have no impact on drivers. Participants have the right to refrain from answering any question within the survey, and this will not affect them in any way possible. Participants will be informed that by agreeing to enroll in this study, they are granting permission for the principal investigator (M Salam) to publish or report their responses at scientific meetings and articles, given their identity will not be revealed. Study investigators will not request any personal identifier during the study conduction. The electronic questionnaires will be coded. Participants’ data will remain anonymous during data entry and analyses. Sociodemographics will be presented in an aggregate form to protect the privacy of participants. To achieve adequate participant enrollment, an incentive will be provided. Since the interview will occur during working hours, we look forward to compensating the PTDs at the end of the survey. The compensation will be a purchasing voucher.

The conduction of the trial will be monitored by the principal investigator (M Salam) and the IRB at the Lebanese American University to ensure the study does not violate the protocol. Initial monitoring will be performed when the first participant is recruited. Random checkups on the informed consent forms and collected surveys will be performed by the principal investigator (M Salam) to ensure the data collectors abide by the study protocol. Weekly meetings with the data collectors will be arranged to make sure the processes of recruiting participants, screening for eligibility, randomization, and survey are well-executed. This study protocol is registered in ClinicalTrials.gov.

## Results

**Figure 1.**
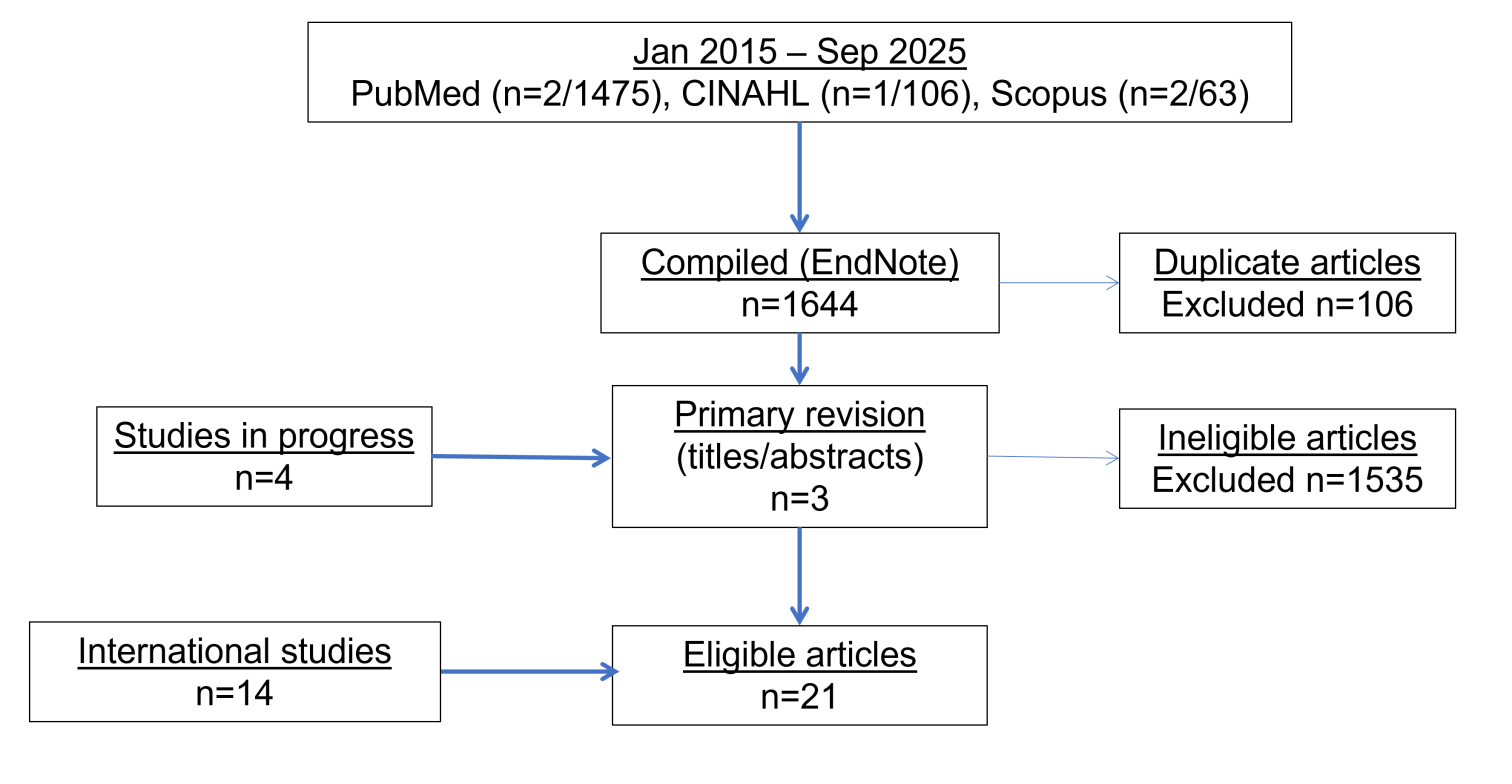
PRISMA (Preferred Reporting Items for Systematic Reviews and Meta-Analyses) flow chart.

Articles retrieved from each of the screened databases were compiled into a reference software manager (EndNote [Clarivate]). Duplicate articles were rapidly identified, confirmed by the research team, and removed. A preliminary review of titles and abstracts was performed by the study investigators. After excluding ineligible articles, the remaining articles were subject to a second round of thorough review (full version) to confirm that these articles met the criteria. In case of a dispute, it was discussed. The extraction of data followed a structured template. Furthermore, authors of published articles were contacted to inquire about studies in progress (M Salam, unpublished data, 2026a; M Salam, unpublished data, 2026b; M Salam, unpublished data, 2026c) [76]. Moreover, international and regional studies were also visited, and information relevant to the context of the study was reviewed (Figure 1).

Based on the review of literature, 3 eligible published studies were identified. A review study of literature on influenza and COVID-19 in Lebanon highlighted the determinants of influenza and COVID-19 vaccine intent or uptake (environmental factors, norms, knowledge, perceptions, attitudes, past experiences, behavioral control, and hesitancy) [48]. An unpublished study identified the challenges that PTDs face in Lebanon (political corruption, hunger, costly health care services, and economic or financial problems; M Salam, unpublished data, 2026). Another study evaluated influenza vaccine hesitancy and its determinants among PTDs in Lebanon. The population characteristics, levels of vaccine hesitancy, and its determinants were presented [77]. Another study (unpublished) reported the prevalence of and factors associated with influenza vaccination uptake among PTDs in Lebanon (M Salam, unpublished data, 2026b). The perceived severity of and susceptibility to COVID-19 among PTDs and commuters after lifting COVID-19 restrictions were investigated. Commuters’ preferences in regard to the transportation system were captured [58]. Another study evaluated risk perceptions to RIDs among university students who use public transportation. Students prefer if PTDs clean or disinfect their vehicles (465/539, 86.3%) and get vaccinated (400/493, 81.1%; M Salam, unpublished data, 2026c). Moreover, 1 study evaluated the cleaning and disinfecting practices of transportation vehicles in Lebanon to control the spread of RIDs (Table 4) [76].

**Table 4.** Summary of relevant literature.

Study	Study objective	Outcome
1: Determinants of influenza and COVID-19 vaccine intent or uptake in Lebanon: a scoping review of the literature [48]	Explored the extent and direction of the literature on influenza and COVID-19 vaccine status in Lebanon.	Determinants of vaccine intent and uptake included environmental factors, norms, knowledge, perceptions, attitudes, past experiences, behavioral control, and hesitancy.
2: Exploring the challenges that public transportation drivers face in Lebanon (M Salam, unpublished data, 2026)	Identified the challenges that public transportation drivers in Lebanon face amid the COVID-19 pandemic, economic collapse, political instability, and armed conflicts	The main challenges were political corruption, hunger, costly health care services, and economic or financial problems.
3: Influenza vaccine hesitancy and its determinants among Lebanese public transportation drivers [77]	Evaluated influenza vaccine hesitancy and its determinants among a representative sample of Lebanese public transportation drivers	The majority of study participants (n=499, 98%) are males; mean age 44.7 (SD 12.6), mean duration of work in public transportation is 11.5 (SD 10.1) y. Mean scores of vaccine hesitancy (1 to 5): risk calculation (mean 3.27, SD 1.28), preference for natural immunity over influenza vaccines (mean 3.23, SD 1.14), and vaccine constraints (mean 2.83, SD 1.38), concerns regarding commercial profiteering behind influenza vaccines (mean 2.77, SD 1.10), and lack of trust in vaccine safety (mean 2.70, SD 1.32).
4: Factors associated with influenza vaccine uptake and intention among public transportation drivers in Lebanon (M Salam, unpublished data, 2026b)	Evaluated the prevalence of and factors associated with influenza vaccination uptake and intention among transportation drivers in Lebanon	Influenza vaccine uptake rate: 28.9%. Influenza vaccine intent: 56.2%, if it was affordable or through a mobile clinic. Factors associated with influenza vaccine uptake: knowing someone who took the vaccine (adjusted OR^a^ 3.62, 95% CI 2.15‐6.10); being advised to take the vaccine (adjusted OR 2.11, 95% CI 1.17‐3.79); and previous influenza infection (adjusted OR 1.93, 95% CI 1.38‐2.70); lack of confidence in vaccines (adjusted OR 0.71, 95% CI 0.59‐0.85); and risk calculation (adjusted OR 0.82, 95% CI 0.68‐0.98). Factors associated with influenza vaccine intention: knowing someone who took the vaccine (adjusted OR 2.44, 95% CI 1.58‐3.75), having an average socioeconomic status (adjusted OR 0.56, 95% CI 0.38‐0.82), concerns about commercial profiteering (adjusted OR 0.73, 95% CI 0.58‐0.92), and a preference for natural immunity (adjusted OR 0.76, 95% CI 0.61‐0.94).
5: COVID-19 risk perceptions among public transportation drivers and users in Lebanon: a cross-sectional study [58]	Evaluated the perceived severity of and susceptibility to COVID-19 among public transportation drivers and users after lifting COVID-19 restrictions in Lebanon	Perceived susceptibility to COVID-19 scores were significantly higher among public transportation users (3.3/5) compared with drivers (3.1/5; *P*=.02). Public transportation users felt at higher risk of getting COVID-19 while using vans or buses (51.5%) compared with taxi cabs (39.4%). Users preferred if drivers disinfect their vehicles (76.5%), preferred sharing a ride with others who wear a face mask (71.4%), preferred if drivers wear masks (69.7%), preferred if the transport vehicle had a protective shield (65.5%), and preferred drivers who get vaccinated against COVID-19 (58.8%). Users disagreed that there is a need for a mandate to vaccinate drivers against COVID-19 (59.6%). Public transportation users not having health insurance, those who wear a face mask during a ride, and being unemployed reported higher perceived severity to or susceptibility toward COVID-19. Among drivers, being married, receiving the COVID-19 vaccine, wearing a face mask, and performing physical activities were associated with higher perceived severity to or susceptibility toward COVID-19.
6: Risk perceptions toward RIDs among university students using the public transportation system in Lebanon (M Salam, unpublished data, 2026c)	Evaluated the perceived severity of and susceptibility to RIDs among university students who utilize the public transportation system	Around 62.1% of students are concerned about the severity of RID^b^, whereas 50.6% feel susceptible to RIDs while using transport vehicles, mainly vans and busses. Students prefer if transport drivers clean or disinfect their vehicles (n=465, 86.3%) and get vaccinated (n=400, 81.1%). Students state that hand sanitizers are unavailable inside vehicles (n=10.5%).
7: Cleaning and disinfecting public transportation vehicles against RIDs: a cross-sectional study [76]	Evaluated the cleaning and disinfecting practices of public transportation vehicles in Lebanon	Noncompliance rates with the frequencies of cleaning, disinfection, and deep cleaning (washing stations) were 296 (75.1%), 321 (81.5%), and 161 (40.9%), respectively. Moreover, 251 (71.9%), 8 (3.4%), and 162 (51.3%) of drivers were not compliant with cleaning, disinfecting, and deep cleaning methods, respectively. Noncompliance with the frequency of changing HVAC^c^ filters was (n=387, 98.2%). 383 (97.2%) and 332 (89.7%) were not compliant with self-protective measures against RID while on duty or while cleaning and disinfecting vehicles, respectively.

a OR: odds ratio.

b RID: respiratory infectious disease.

c HVAC: heating, ventilation, and air conditioning.

In total, 14 studies from international literature were visited. These studies reported the global rates and disease burden of RIDs, the benefits of vaccination to individuals, the community, and health care systems [12,60,61,78]. Evidence from the US National Institute for Occupational Safety and Health regarding PTDs being a high-risk occupational group will be included in the video [7,16-18]. The risk of RID transmission in public transportation vehicles reported in 1 study will be presented in the video [8]. The main outcomes in other studies are presented in Table 5.

In stages 2 and 3, the video will be revised by experts (projected 5 months), produced (projected 6 months), and tested (projected 2 months). The timeline for this project is described in Table 6.

**Table 5.** Summary of additional studies.

Study	Study finding and reference
1	Every year, 3-5 million individuals worldwide get severely sick because of influenza. Between 250,000 and 500,000 individuals pass away yearly [2].
2	Vaccination is an essential primary health care objective with benefits at the individual, health care system, and community levels. Vaccination allows for an expedited health recovery, fewer visits to clinics, less stress on health care systems, and lower medical bills [59].
3	Vaccination reduces the risk of contracting the virus by shortening the duration of viral infectivity and transmission. It reduces disease severity and mortality, especially among vulnerable groups [12].
4	A wide vaccine coverage promotes efficient herd immunity and subsequently fewer disease outbreaks [60,61].
5	As per the US NIOSH^a^, there is ample evidence indicating that public transportation drivers are at high risk of contracting influenza [7,16-18].
6	Public transportation involves crowdedness, unsanitary conditions, and close or prolonged interaction with many passengers throughout the day [6,7].
7	The risk of influenza transmission in passenger cars was reported to range between 59% and 99.9% for a 90-min trip [8].
8	In the 2017‐2018 influenza season, work absenteeism (1.7%‐3.6%) among US drivers due to influenza exceeded that witnessed during the previous 5 years [57].
9	The real concern is when drivers who contract influenza become potential “super-spreaders” of influenza infection, which makes the broader community of commuters and their social network at risk of exposure to influenza [9].
10	The MoPH^b^ in Lebanon reported that, in February 2020, the influenza transmissibility was moderate, yet it had a very high impact. A sentinel surveillance of positivity rate testing (captured from 8 Lebanese hospitals between 2018 and 2020) showed that 30%‐40% of severe acute respiratory infections (n=1238) were due to influenza viruses, mainly Influenza A(H1N1)pdm, Influenza A(H3N2), and Influenza B Victoria [79].
11	Between 41% and 43% of influenza cases in Lebanon were associated with severe acute respiratory infections. The seriousness of influenza infections was also notable, with 20‐50 deaths per week by mid-February of 2020 [80].
12	Influenza vaccine uptake rates in Lebanon are estimated at less than 20 doses per 1000 people [81].
13	The Lebanese MoPH stated that the country witnessed more than 1 million confirmed cases of COVID-19 as of February 2022, among whom more than 10,000 people died [82].
14	Drivers’ adherence to COVID-19 precautionary measures can boost the commuters’ satisfaction if they feel safe from COVID-19 during the ride [39].

a NIOSH: National Institute for Occupational Safety and Health.

b MoPH: Ministry of Public Health.

**Table 6.** Timeline of the study.

Phases and tasks	Involvement duration (fiscal months)											
	May	Jun	July	Aug	Sep	Oct	Nov	Dec	Jan	Feb	Mar	Apr
First year (2026)												
Communications or approvals (IRB^a^)	✓											
Inviting panel of experts		✓										
Circulating the script (round 1)			✓									
Circulating the script (round 2)				✓								
Circulating the script (round 3)					✓	✓						
Developing the video							✓	✓	✓	✓	✓	✓
Second year (2027)												
Translation of tool – pilot test	✓											
Trial		✓	✓									
Analysis				✓								
Writing of manuscript					✓	✓						
Publication							✓	✓	✓	✓	✓	✓

a IRB: institutional review board.

## Discussion

### Principal Findings

This multistage study is initiated by a scoping review of literature to identify articles on RID among PTDs and draft a script for a health-awareness video on precautionary measures against RIDs. Future publications with similar objectives will be considered before the revision of the script. The script will be scientifically revised by a panel of experts to ensure it is evidence-based, culturally oriented, socially acceptable, and tailored toward the target viewers. The PEMAT-AV and DICERN tools are valid and reliable tools to evaluate the script. Scores obtained in this study will be compared with those produced by similar studies if available. The effectiveness of the video produced is scrutinized by testing its impact on knowledge, risk perceptions, and attitudes among a random sample of PTDs. This controls for selection bias and the effect of confounding variables; thus providing a robust statistical foundation.

Evidence-based health awareness videos are cost-effective and disseminate credible information. Designing video content that is guided by robust theoretical frameworks maximizes the efficacy of health promotion [24]. Raising awareness elevates health-risk perceptions, triggers CD, and enhances attitudes [44-46]. RID perceived severity is how PTDs feel about the seriousness of RID, or what could happen if precautionary measures against RIDs are not taken. Perceived susceptibility is their feeling about the probability of contracting RID or encountering its undesirable outcomes [83]. Any risk miscalculation indicates complacency. Exposure to the video content is likely to trigger CD among PTDs. They will avoid or ignore uncomfortable feelings using various forms of dissonance reduction strategies. Dissonance-based interventions are known to be effective in changing various health-related behaviors through risk reduction [84]. PTDs will seek advice and guidance, and it will be offered to them at the end of the video.

Findings in this study will have implications for advancing knowledge and practice. The health-awareness video will enhance the knowledge of PTDs on RIDs and its precautionary measures, including the usage of facemasks and vaccination. PTDs will be more vigilant about cleaning and disinfecting their vehicles. Adherence to RIDs and precautionary measures contributes to higher service satisfaction and subsequently higher usage of the transportation system. Using the public transportation systems reduces street congestion, controls air pollution, saves money for users, and generates profits for stakeholders. Promoting a safe public transportation system reflects positively on public health, aligns with the CDC mission to control the global spread of diseases, and lessens disease burden.

A few limitations are anticipated in this study. The search strategy in the scoping review is limited to one country, which might limit the generalizability of findings elsewhere. It is designed as such to ensure the characters and narrative in the video resemble those of the target population. Reviews are prone to potential bias, so every effort was made to control them. Furthermore, 2 investigators (M Salam and MH) revised all eligible articles, thus eliminating any chance of committing evidence selection bias. Critical appraisal of articles passed through 2 sequential stages. The quality of the screened journals was examined. PTDs are an underresearched, so more evidence was sought from international literature. Regarding the Delphi study, some reviewers of the script might not respond promptly, and it might delay the study progress. Frequent reminders and meetings with them can be arranged to ensure timeliness. Regarding the trial study, the nature of PTDs’ job makes it difficult to survey them while on duty. The research team has previous experience with this population, who are often compensated for their time lost on duty.

### Conclusions

An evidence-based health awareness video on precautionary measures against RIDs tailored toward PTDs contributes to a safer public transportation system and higher service usage. It aligns with the CDC mission to control the global spread of RID and lessen disease burden. Study findings will be shared with key stakeholders, such as the Lebanese Ministry of Public Health, syndicates, and policymakers. Their contact information and help desks will be enlisted at the end of the video for future consultation. Stakeholders are expected to widely circulate this video through their official and social media channels. They will be asked to support the affordability, availability, and accessibility of PTDs to personal protective equipment (facemasks, hand sanitizers, vehicle disinfectant products, and vaccines). Between groups analysis will indicate the effectiveness of the video, and within group analysis will identify PTDs who are more complacent and resistant to precautionary measures against RIDs. In the absence of mandates to wear facemasks or take vaccines, raising awareness remains a key primary health initiative.

## Supplementary material

10.2196/89474 Checklist 1 PRISMA-ScR fillable checklist.

10.2196/89474 Checklist 2 DELPHISTAR checklist.

10.2196/89474 Checklist 3 SPIRIT checklist.

## Abbreviations

CD: cognitive dissonance
CDC: Centers for Disease Control and Prevention
HBM: Health Belief Model
IRB: Institutional Review Board
PEMAT-AV: Patient Education Materials Assessment Tool for audiovisuals
PMS: Percentage Mean Score
PRISMA-ScR: Preferred Reporting Items for Systematic reviews and Meta-Analyses extension for scoping reviews
PTD: public transportation driver
RID: respiratory infectious disease
SPIRIT: Standard Protocol Items: Recommendations for Interventional Trials
VAX: Vaccination Attitudes Examination
