# Supplementary material for: Production of a Health Awareness Video on Precautionary Measures Against Respiratory Infectious Diseases for Public Transportation Drivers: Protocol for a Scoping Review, a Delphi Study, and a Randomized Controlled Trial
Source: JMIR Res Protoc. 2026 Jun 19;15:e89474. doi: 10.2196/89474 (PMC13284516; doi:10.2196/89474)
Supplement: Checklist 2 [file resprot-v15-e89474-s002.pdf]

DELPHISTAR

Delphi studies in social and health sciences – recommendations for a standardized reporting

DeWiss Delphi techniques in health and social sciences

PH Schwäbisch Gmünd University of Education

Delphi studies in social and health sciences – recommendations for an interdisciplinary standardized reporting (DELPHISTAR).

From: Niederberger, M., Schifano, J., Deckert, S., Hirt, J., Homberg, A., Köberich, S., Kuhn, R., Rommel, A., Sonnberger, M. & the DEWISS network (2024). Delphi studies in social and health sciences—Recommendations for an interdisciplinary standardized reporting (DELPHISTAR). Results of a Delphi study. *PLoS ONE 19(8):* e0304651. https://doi.org/10.1371/journal.pone.0304651

More information under OSF (https://osf.io/gc4jk) and DEWISS (https://delphi.ph-gmuend.de/)

What is the aim of DELPHISTAR?

- Improve, harmonize, and make the reporting in publications on Delphi studies comparable
- Facilitate the evaluation of Delphi studies including during peer review processes
- Reduce, and ideally prevent, inconsistencies and unclear descriptions in publications on Delphi studies
- Raise awareness of the diversity among the Delphi variants and of their specific potentials and challenges

DELPHISTAR is a Delphi reporting guideline that is:

- valid for all Delphi variants (e.g., classic Delphi, real-time Delphi, group Delphi, policy Delphi, argumentative Delphi, café Delphi)
- applicable to different purposes (e.g., Delphi studies to establish consensus, to gather expert judgments or to forecast)
- given equal consideration in the health and social sciences

This reporting guideline is meant for studies using Delphi techniques in the health and social sciences. These also include all Delphi variants and modifications that meet the following criteria:

1. Survey of several people (=experts) with specialized knowledge (e.g., operational knowledge, experiential knowledge, functional knowledge, contextual knowledge)
2. Structured communication process
3. Carrying out at least two survey rounds or the option to respond at least two times
4. Feedback: the (interim) results are presented to the experts starting from the second round
5. Basis is a quantitative questionnaire with the possibility to contribute or supplement arguments for the respective position
6. Quantitative and qualitative answer are systematically analyzed (quantitative: e.g., descriptive statistics, qualitative: e.g., thematic analysis)

This reporting guideline is available in English and German at https://delphi.ph-gmuend.de/activities/delphistar (last update October 2024).

Contact

Prof. Dr. Marlen Niederberger
E-mail: marlen.niederberger@ph-gmuend.de
Department of Research Methods in Health Promotion and Prevention
Institute for Health Sciences, University of Education Schwäbisch Gmünd,
Oberbettringer Straße 200, 73525 Schwäbisch Gmünd, Germany

Delphi studies in social and health sciences – recommendations for an interdisciplinary standardized reporting (DELPHISTAR)

Topic	Section	Item	Checklist Item	Location where item is reported	Exemplary wording
I<br>Title and Abstract		1	Identification as a Delphi study in the title	P1	Production of a Health Awareness Video on Precautionary Measures Against Respiratory Infectious Diseases for Public Transportation Drivers: A scoping review, a Delphi study, and a Randomized Controlled Trial
		2	Identification as a Delphi study in the abstract	P2	Using a classic Delphi technique, script will be revised and approved by eight experts
		3	Structured abstract	P2	Background; Objective; Methods; Results; Conclusions
II<br>Context	Formal	4	Information about the sources of funding	NA	
		5	Information about the team of authors and/or researchers (e.g., discipline, institution)	P2,6	Script will be revised and approved by eight experts in public health, infection control, psychology, and filmmaking
		6	Information about method consulting	P9	Based on the findings of the review, a script for a health-awareness video will be drafted. Using a classic Delphi technique, the script will be revised and approved.
		7	Information about the project background	P7	This multi-stage project will be executed over two fiscal years. In Stage one, a scoping review study will identify articles on RID among PTDs. In Stage two, based on the findings of the review, a script for a health-awareness video will be drafted. Using a classic Delphi technique, the script will be revised and approved by eight experts in public health, infection control, psychology, filmmaking, and public transportation. A video is then produced based on the approved script. In Stage three, a non-blinded, randomized, two-arm controlled trial will be conducted on 387 PTDs to evaluate the effectiveness of the video.
		8	Information about the study protocol	NA	
	Content	9	Justification of the chosen method (Delphi) to answer the research question	P11	The script will be revised by a panel of experts in public health, infection control, psychology, filmmaking, and public transportation (drivers, users, stakeholders). The expert in public health will ensure the script promotes good knowledge among viewers. The expert in infection control and prevention will advise on information regarding RID transmission, precautionary measures, risk factors, and vulnerable populations. The expert in psychology will emphasize on the details in the script that would elevate health-risk perception, encourage positive attitudes, and influence

Topic	Section	Item	Checklist Item	Location where item is reported	Exemplary wording
		11			behavioral changes. The expert in filmmaking will advise on the general plot, characters, duration and the narrative text so that the health-awareness video leads to a meaningful impact among the target audience.
		10	Aim of the Delphi study (e.g., consensus, forecasting)	P6	Experts will objectively evaluate the script using the Patient Education Materials Assessment Tool – Audiovisual (PEMAT-AV) and DICERN tools. Additional subjective comments will be accommodated.
III Method	Body & Integration of knowledge	11	Identification and elucidation of relevant expertise, spheres of experience, and perspectives (e.g., theory, practice, affected groups, disciplines)	P9	The expert in public health will ensure the script promotes good knowledge among viewers. The expert in infection control and prevention will advise on information regarding RID transmission, precautionary measures, risk factors, and vulnerable populations. The expert in psychology will emphasize on the details in the script that would elevate health-risk perception, encourage positive attitudes, and influence behavioral changes. The expert in filmmaking will advise on the general plot, characters, duration and the narrative text so that the health-awareness video leads to a meaningful impact among the target audience. A PTD will be involved in the review of the script to ensure the script mimics the real-life experience of drivers.
		12	Handling of knowledge, expertise and perspectives which are missing or have been deliberately not integrated	P9	In case one of the members did not respond at any round, the expert will be sent timely reminders. If non-response persists, the expert will be replaced by another expert with the same specialty. To encourage participation, the experts will be acknowledged in the future publication and video production. Non-responses will be evaluated at each round to make sure the study objectives are still achievable.
		13	Basic definition of expert[1]	P9	A purposive sampling method will be followed to identify these experts. The panel members will be reached out through their professional networks or based on their publication profiles, if they are experts in their field for at least 5 years.
	Delphi variant and modifications	14	Identification of the type of Delphi variant and potential modifications (e.g., classic Delphi, real-time Delphi, group Delphi)	P9	Using a classic Delphi technique, the script will be revised by a panel of experts

Topic	Section	Item	Checklist Item	Location where item is reported	Exemplary wording
		15	Justification of the Delphi variant and modifications, including during the Delphi study, if applicable	P9	At least three rounds of review will be performed after circulating the script to all members of the panel. The first round will focus on exploring the characters, role plays, settings and plot. Experts will evaluate the quality of health information integrated in the script. Suggested modifications will be carried out before the next round. If panel members have additional recommendations, it will be integrated in the script and a third round of review will follow. In case one of the members did not respond at any round, the expert will be sent timely reminders. If non-response persists, the expert will be replaced by another expert with the same specialty. To encourage participation, the experts will be acknowledged in the future publication and video production. Non-responses will be evaluated at each round to make sure the study objectives are still achievable
	**Sample of experts**	16	Selection criteria for the experts (per round, per expert group if applicable)	P11	
		17	Identification of the experts	P9	A purposive sampling method will be followed to identify these experts. The panel members will be reached out through their professional networks or based on their publication profiles, if they are experts in their field for at least 5 years.
		18	Information about recruiting and any subsequent recruiting of experts	P9	The experts will be informed about the Delphi study and invited to participate.
	**Survey**	19	Elucidation of the content development for the questionnaire[2]	P6	Experts will objectively evaluate the script using the Patient Education Materials Assessment Tool – Audiovisual (PEMAT-AV) and DICERN tools. Additional subjective comments will be accommodated.
		20	Description of the questionnaire (content and structure)	P9-10	PEMAT-AV systematically evaluates the understandability and actionability of educational materials. DISCERN facilitates the production of new, high quality, and evidence-based consumer health information
		21	Number of Delphi rounds	P10	Three Delphi rounds will be held.

Topic	Section	Item	Checklist Item	Location where item is reported	Exemplary wording
	**Delphi rounds**	22	Information about the aims of the individual Delphi rounds	P10	The first round will focus on exploring the characters, role plays, settings and plot. Experts will evaluate the quality of health information integrated in the script. Suggested modifications will be carried out before the next round. If panel members have additional recommendations, it will be integrated in the script and a third round of review will follow.
		23	Disclosure and justification of the criterion for discontinuation	P11	Three or more rounds of review if needed
	**Feedback**	24	Information about what data was reported back per round	NA	
		25	Information on how the results of the previous Delphi round were fed back to the experts surveyed (e.g., via frequencies, mean values, measures of dispersion, listing of comments)	NA	
		26	Information on whether feedback was differentiated by specific groups (e.g., by field of expertise, institutional affiliation)	NA	
		27	Information about how dissent and unclear results were handled	NA	
	**Data analysis**	28	Disclosure of the quantitative and qualitative analytical strategy	P11	PEMAT-AV and DISCERN scores will be obtained from each member of the panel, at every round of the review. For the PEMAT-AV scores, statistically significant changes in the understandability and actionability scores indicates that the quality of the script is enhanced with each round. The scores will be presented in means±standard deviation(SD). For the DISCERN, scores will be converted to low, moderate and high ability of the script to clearly deliver the message on precautionary measures against RID to viewers..
		29	Definition and measurement of consensus	P11	Change in scores indicate that the quality of the script is improving across the rounds of review, the decision to produce the video is based on the final approval of the panel members.

Topic	Section	Item	Checklist Item	Location where item is reported	Exemplary wording
		30	Information on group-specific analysis or weighting of experts (e.g., theory vs. practice, discipline-specific analysis)	P11	Paired t-test (for two rounds of review) and repeated measures analysis of variance (for three or more rounds of review if needed) will be performed if the conditions of normality are met. Otherwise, Wilcoxon Signed-Rank Test and Friedman Test will be used for the two round or three round review, respectively. DISCERN ordinal outcomes will be presented in frequencies and percentages for each round of review, and Cochran's Q test will be used to detect improvements in the clarity of the script across the review rounds
IV Results	Delphi process	31	Illustration of the Delphi study (e.g., in a flow chart)	NA	
		32	Information about special aspects during the Delphi study (e.g., deviations from the intended approach with justification)	NA	
		33	Number of experts per round (both invited and participating)	NA	
	Results	34	Presentation of the results for each Delphi round and the final results	NA	
V Discussion	Quality of findings	35	Highlighting the findings from the Delphi study	NA	
		36	Validity of the results (e.g., transferability of the findings)	NA	
		37	Reliability of the results (e.g., split half, inter-rater reliability)	NA	
		38	Reflection on potential limitations (e.g., number of experts, response bias)	NA	

[1] "Experts" are the participants; these can be people from academia, practice, or representatives of lived experience (e.g., patients, family members).
[2] The term "questionnaire" stands for the survey instrument regardless of whether quantitative or qualitative items are integrated or weighted.
